# The legal guardians' dilemma: Decision making associated with invasive non-life-saving procedures

**DOI:** 10.1186/2045-4015-1-36

**Published:** 2012-09-24

**Authors:** Michael Kuniavsky, Freda DeKeyser Ganz, David M Linton, Sigal Sviri

**Affiliations:** 1 General ICU, Asaf HaRofeh Medical Center, Beer Jaacov 70300, Israel; 2 Medical ICU Hadassah-University Medical Center, Jerusalem, Israel; 3 Hadassah-Hebrew University School of Nursing, Faculty of Medicine, Jerusalem, Israel; 4 The Hebrew University Hadassah School of Public Health and Community Medicine, Faculty of Medicine, Israel

**Keywords:** ICU, Legal guardian, Decision making, Invasive procedures, Relatives, Tracheotomy

## Abstract

**Background:**

ICU patients frequently undergo non-life-saving invasive procedures. When patient informed consent cannot be obtained, legal guardianship (LG), often from a close relative, may be required by law. The objective of this cohort study was to investigate the attitudes of LGs of ICU patients regarding the process of decision making for invasive non-life-saving procedures.

**Methods:**

The study was conducted from May 2009 until June 2010 in general medical/surgical ICUs in two large Israeli medical centers. All 64 LGs who met the study criteria agreed to participate in the study. Three questionnaires were administered: a demographic data questionnaire, the Family Satisfaction with ICU 34 Questionnaire, and the Attitudes towards the LG Decision Making Process questionnaire, developed by the authors.

**Results:**

The sample consisted of 64 LGs. Most participants were married (n = 56, 87.5%), male (n = 33, 51.6%), who had either a high school (n = 24, 37.5%) or college (n = 19, 29.7%) education, and were at a mean age of 49.2 (±11.22). Almost all of the procedures performed were tracheotomies (n = 63, 98.4%). About two-thirds of the LGs preferred decisions to be made by the medical staff after discussing options with them (n = 42, 65.6%) and about three-fifths stated that decisions could be made without the need for the appointment of an LG (n = 37, 57.8%). Attitudes towards ease of obtaining information and honesty of information were more positive compared to those of consistency and understanding of information.

**Conclusions:**

The legal guardianship process requires better communication and more understandable information in order to assist LGs in making decisions for others in at times vague and stressful situations.

## Background

ICU patients often require invasive procedures, but consent is often difficult to obtain because patients are either unconscious or sedated [1-4]. In life saving situations, such as emergency surgery, patient consent may be waived if unobtainable. However, in non-life-saving procedures, such as tracheotomy and percutaneous endoscopic gastrostomy insertion, procedures that are considered standard care for long-term ventilated patients who are unable to undergo extubation, patient consent is often required and, if unavailable, relatives are often required to be involved in the decision-making process. These decisions are made worldwide via different decision-making processes, ranging from a paternalistic model (where physicians make all of the decisions) to an autonomous model (where the patient and/or family make all of the decisions) [5-8]. In some countries the appointment of a legal guardian (LG) may be required by law for all decisions related to non-life-saving invasive procedures. The LG is often a first degree relative who is then authorized to make the decisions and signs the informed consent form.

In Israel, the family has no legal standing to make medical decisions for the patient unless they are appointed as legal guardians or have power of attorney from the patient for bodily issues. There are therefore a number of legal options for performing invasive procedures when a patient is unable to give informed consent. In cases of life-threatening emergencies (e.g., emergency surgical procedures that have to be performed within 24 hours), the Patient Rights Law (1996) states that three physicians' signatures are required in order to perform the procedure without the patient's consent. However, if any one or more of five interventions are indicated (non-life saving surgical procedures, angioplasty, dialysis, radiotherapy, chemotherapy, and *in vitro* fertilization), consent is obtained from a relative who has power of attorney or is a legal proxy of the patient for bodily issues; if none are available, a legal guardian must be appointed for the patient by the courts, in order to give a written informed consent for the intervention on behalf of the patient. In this situation, the legal guardian is actually acting as a legal stand-in for the patient. This relates to understanding the implications of the decision, including any undesired side-effects or complications, but does not absolve the clinician of any negligence liability.

Other legal options associated with non-life-threatening situations are based on the Patient Rights Law (1996) and Law of the Dying Patient (2005). According to these laws, any legally competent person may formulate advanced medical directives or appoint a family member to have power of attorney to make medical decisions on their behalf in the future. Patients and/or their proxies must receive full information from a health care provider (physician or nurse) prior to making a medical decision, and this decision is honored by the medical team. Unfortunately, in Israel as well as other countries, advanced directives are not performed often, and the patient's views and attitudes are often not clear to the family and to the treating medical team [9,10]. In cases when the patient cannot give his or her consent, and no advanced directives or power of attorney are in place or applicable, the law requires the appointment of a legal guardian (LG) in order to obtain informed consent for non-life-saving invasive procedures on behalf of the patient. This option is currently widely practiced in ICUs in Israel. Usually the LG is a close relative or relatives, chosen and agreed upon by the rest of the family. The appointment is made by the Court who reviews recommendations made by the attending physician and a social worker after discussions with the family and is valid for three months. Following the appointment, the LG is legally entitled to make decisions regarding any treatments including invasive procedures for the patient. It should be stressed that in cases in which advanced directives or power of attorney had been obtained, the patient had expressed his or her wishes regarding treatment in certain clinical situations, a fact that helps direct the family and medical team towards a more representative decision of the patient's wishes.

However, in most cases of LG appointment, explicit discussions with the patient had not taken place and their wishes are not always clear. The LG is actually the representative of both the patient and the family. As such, the LG is exposed to a high level of anxiety, as he or she is responsible for decisions related to performing or withholding treatments and procedures. These feelings are often exacerbated by the fact that the patient's wishes regarding medical interventions in general, as well as the specific intervention being considered, are often unknown to the LG and other family members. Sviri et al. found that 67% of family members of chronically ventilated patients have not discussed their preferences with their family members [9]. This situation is even more complex in ICU settings, where patients are acutely rather than chronically ill.

Family members of ICU patients often experience difficulty making decisions for their critically ill relatives [10]. Many of them experience anxiety and depression while their relative's life is at stake [11]. Family members are at times incapable of fully understanding medical explanations, especially regarding their relative's treatment options and prognosis [10,11]. Decision making in these situations often increases the burden on family members. It is therefore not surprising that more than half of the respondents in Azoulay et al.'s study preferred not to participate in the decision-making process [3], while in Canada, 81% of the respondents preferred to share the decision making with the medical staff and only 19% preferred to make the final decision by themselves [4].

The complexity of legal guardianship for ICU patients in Israel (as opposed to the more general issue of family involvement in patient care), although quite common in the ICU setting, has not been extensively studied. The attitudes of family members regarding the legal guardianship process and decision making for invasive non-life-saving procedures have not been widely investigated. Research on LGs in Israel is limited and no studies of LGs have been carried out in Israeli ICU settings [12].

In the current study, we looked at the processes of obtaining legal guardianship from the family in non-life-threatening situations in Israel. The aim of the current study was to examine the attitudes of LGs of ICU patients towards the decision making process for their critically ill relatives.

## Methods

### Population and sample

The study was conducted from May 2009 until June 2010 in the medical/surgical ICUs of two large Israeli medical centers following approval by the local Institutional Review Boards. Inclusion criteria were LGs of ICU patients who could not provide informed consent and required non-life-saving invasive procedures and who were able to complete a questionnaire in the Hebrew language.

All LGs who met the study criteria during the data collection period were asked to participate in the study and all agreed to participate. A total of 64 LGs were recruited into the study, 32 from each center. The sample size was based on a power analysis where the level of power was set at 0.8 with an alpha level of 0.05, and a weak-moderate effect size. A pilot study of 10 subjects was performed.

### Instruments

Three questionnaires were presented to the study sample: a demographic questionnaire; the Family Satisfaction in ICU 34 (FS-ICU 34) questionnaire, and the Attitudes towards the LG Decision Making Process questionnaire (ADMAP), developed by the authors. The demographic questionnaire contained demographic data (age, gender, religion, religiosity, level of education) of the respondents and the patients, and their relationship to one another. The FS-ICU 34 was originally developed by Heyland and Tranmer [13]. The section regarding family satisfaction with decision making for critically ill patients was used in the study after backward-forward translation to Hebrew (with permission). The section contains two components: "information" and "decision making" needs. The "information" needs component is composed of five questions. The Cronbach reliability for this section in our study was found to be 0.92. The "decision making" component contains 16 items and a Cronbach’s α of 0.78 was obtained. In order to compare the components of the questionnaire, indices of each component were converted to a scale from 0 (least satisfied) to 100 (most satisfied), as described by the developers (Heyland & Tranmer [13]). The third questionnaire, Attitudes of LG regarding the Decision Making Process (ADMAP), was developed by the authors in order to determine the attitudes of LGs towards the guardianship process in Israel (Additional file 1). The tool included nine closed items using a Likert-type scale, ranging from 1 (strongly disagree) to 5 (strongly agree). Content validity was confirmed by 15 ICU physicians, nurses, and social workers. Cronbach’s α reliability was evaluated for the pilot study (N = 10) and for the final sample (N = 64) and was found to be 0.82 and 0.64, respectively.

### Data collection

The LGs appointed by the Court, as required by law, received an explanation of the procedure and signed the informed consent form as required. The anonymous study questionnaires were provided to the LG by the researcher (MK) after receiving an explanation about the study. The questionnaires were completed by the LGs in the family waiting area, while the investigator was available nearby for any questions or clarifications if needed.

Data analysis was performed using the SPSS 14 statistical package. Descriptive statistics were performed for demographic data: frequencies, measures of central tendency, and variance were analyzed. In order to determine associations between variables we used the Pearson Product Moment Correlation. For determination of differences of means between groups for continuous variables t-tests were performed. Analysis of the FS-ICU 34 questionnaire was performed as described by the developers [13].

## Results

All of the respondents in the study were LGs who were appointed by the Court. In none of the cases were advanced medical directives or authorized powers of attorney made prior to admission. Almost all of the invasive procedures were tracheotomies (n = 63, 98.4%).

Demographic data of patients and LGs are provided in Table1. The mean age of the LGs and the patients was 49.2 (±11.22) and 64.9 (±18.68) years, respectively. Most of the LGs were either the children (n = 35, 54.7%) or partners (n = 13, 20.3%) of the patients. All LG appointments took place during the current hospitalization and only 2 (3.1%) had previously been appointed as LGs. In all the cases, all of the family members agreed to the LG appointment. More than 40% of the respondents lived with the patient (n = 27) prior to admission. Of those who did not live with the patient, more than 90% (n = 32, 91.4%) met with them at least once a week prior to their hospitalization.

**Table 1:** Demographic data

	**Patient data (N = 64)**		**LG data (N = 64)**	
	**N**	**%**	**N**	**%**
Male	36	56.3	33	51.6
Female	28	43.7	31	48.4
Single	8	12.5	6.3	4
Married	34	53.1	87.5	56
Divorced	4	6.2	6.2	4
Widowed	18	28.2	-	-
**No of children**	3.2^c^ (2.49±)^a^	2.9^b^ (2.04±)^a^		
Jewish	61	95.3	61	95.3
Muslim	1	1.6	1	1.6
Christian	2	3.1	2	3.1
Religious	19	29.7	18	28.1
Conservative	23	35.9	23	35.9
Secular	20	31.3	21	32.8
Other	2	3.1	2	3.1
Grade school	21	32.8	3	4.7
High school	21	32.8	24	37.5
College	10	15.6	19	29.7
BA	7	10.9	9	14
MA +	4	6.3	9	14
Spouse			13	20.3
Parent			11	17.2
Sister/brother			5	7.8
Son/daughter			35	54.7

^a^average and (SD).

^b^data not available from 3 (4.7%) respondents.

^c^data not available from 5 (7.8%) respondents.

**Figure 1:**
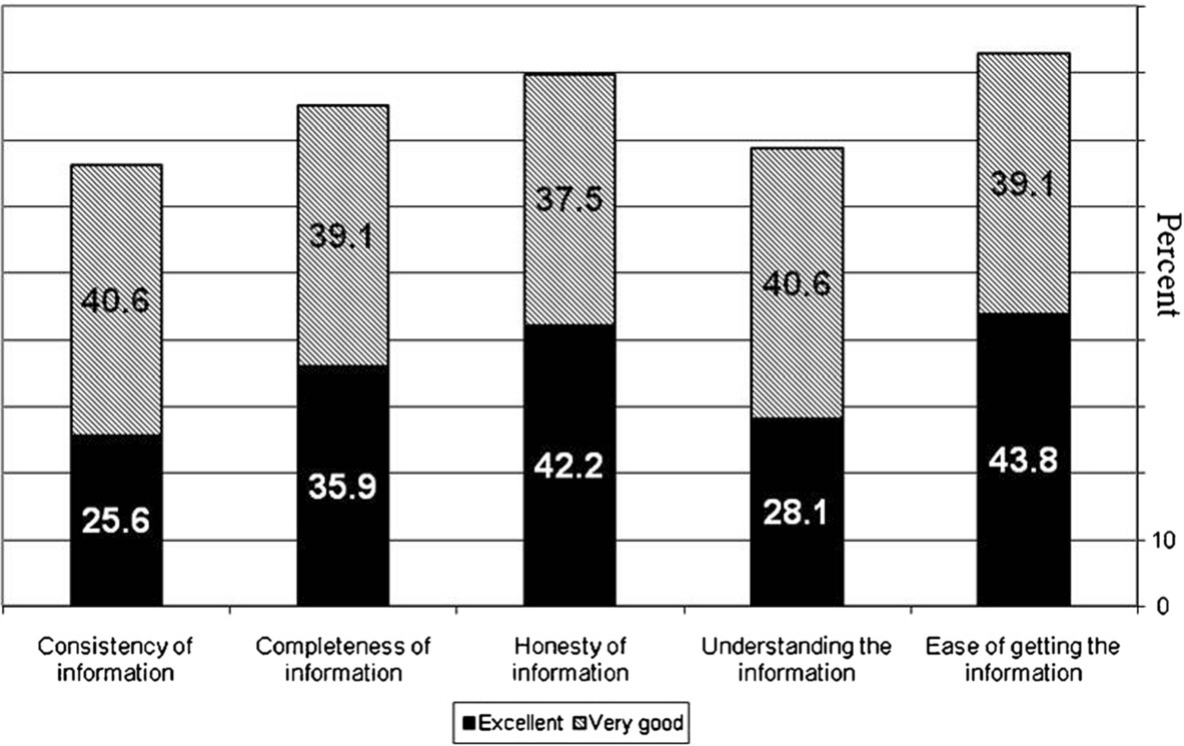
FS ICU 34, "information needs" section (questions 1-5); percentage of LGs who answered "excellent" or "very good" for each question.

The FS-ICU 34 "information" needs section showed that LGs were generally satisfied with the information they received from the medical staff, as most answered "excellent" or "very good" in all questions in this section (Figure1). However, “information consistency” (n = 42, 66.2%) and “understanding the information” (n = 44, 68.7%) received lower satisfaction scores compared to other items such as “ease of getting information” (n = 53, 82.9%) and “honesty of information” (n = 51, 79.7%). See Figure1.

The decision making section of the FS-ICU 34 revealed that more than half of the LGs (n = 37, 57.8%) did not feel they had control over the care of their family member. The majority of respondents felt somewhat (n = 33, 51.6%) or very (n = 22, 34%) involved in the decision-making process. Over three-quarters of the respondents felt they were involved at the right time (n = 50, 78.1%), received the appropriate amount of information to participate in the decision making process (n = 56, 87.5%), had enough time to think about the information provided (n = 53, 82.8%), and had enough time to ask questions and to express their concerns and fears (n = 54, 84%). About three-quarters of the LGs felt either supported (n = 23, 35.9%) or greatly supported (n = 24, 37.5%) during the decision-making process. Most subjects (n = 40, 62.5%) felt that they were given an adequate amount of hope for the recovery of the patient. About two-thirds of the respondents (n = 44, 68.8%) reported agreement within the family regarding the care of the patient. Almost all the LGs (n = 63, 98.4%) were satisfied with the level of healthcare that the patient received, with 43.8% (n = 28) completely satisfied, 32.8% (n = 21) very satisfied, and 21.9% (n = 14) mostly satisfied. Most of respondents (n = 58, 90.7%) were satisfied with their role in the decision-making process, with 39.1% (n = 25) mostly satisfied, 32.8% (n = 21) very satisfied, but only 18.8% (n = 12) completely satisfied. A significant correlation was found between the FS-ICU 34 "information needs" and the "decision making" sections (*r* = 0.58, *p* < 0.001).

**Figure 2:**
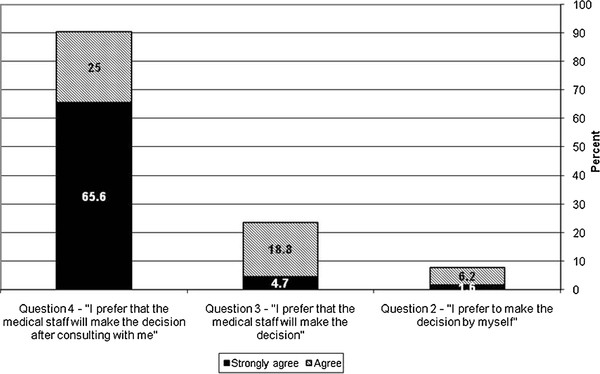
LGs who "agreed" or "strongly agreed" to questions regarding the decision-making process in the ADMAP questionnaire.

The results of the ADMAP questionnaire showed that about two-thirds of the LGs preferred that decisions be made by the medical staff after discussing options with them (n = 42, 65.6%, Figure2). Almost all of the respondents (n = 60, 94.1%) reported that all of the family members agreed to perform the invasive procedure. The majority of respondents (n = 55, 85.9%) reported that they would not change their decision regarding the invasive procedure even if they were not appointed as LGs. Over half of the respondents felt that the decision could have been reached without the need for an LG appointment (n = 37, 57.8%). Almost two-thirds of the respondents (n = 42, 65.6%) stated that they would not consider suing the medical staff even if the invasive procedure was performed without their consent. Just under half of the LGs (n = 30, 48.6%) were aware of the patients’ preferences regarding invasive procedures, and 37.5% (n = 24) of respondents weren’t sure. Just over half of the respondents (n = 34, 53.1%) stated that they took their relative's preferences into consideration when making decisions regarding the invasive procedure. Results of the ADMAP questionnaire are presented in Additional file 1.

No significant associations were found between the main study variables and socio-demographic variables such as LG gender, LG-patient relationship, LG and patient education and age, patient living with the LG, LG number of children, and the reason for the current hospitalization.

## Discussion

Our study has shown that LG appointment was supported by other family members and almost all of the invasive procedures that required LG appointment were tracheotomies. LGs were generally satisfied with the information they received and with the decision-making process. This satisfaction was not correlated with any demographic characteristics of the LG or of the patient. However, LGs were less satisfied with the level of consistency and with the level of understanding of the information they received.

Our findings are consistent with the questionnaire developers' previous findings [4,13,14] and with those of Pochard et al. [11]. Respondents were most satisfied with "Ease of getting information" both in our study and in Heylands et al.'s [4]. However, Heylands et al. did not find lower satisfaction with the "understanding of information” [4].

According to the findings of this study, LGs receive a lot of information from the medical staff that is not always consistent and fully understood. The main reasons cited in the literature for these findings are incoherent and contradictory information and inadequate communication between the medical staff and the family. This information is presented under conditions of stress, anxiety, and confusion, further decreasing the ability of families to understand complex information [3,11,15,16]. This situation is exacerbated by the fact that family conversations are often conducted by different medical staff members, without clear guidelines for such discussions. This could cause different information to be given, sometimes conflicting with previous information as understood by the family, thereby causing confusion [11,17]. In view of our findings, there is a need to improve the quality of the information communicated to families. Staff members should be concerned with providing both consistent and complete information regarding the patient’s current and expected future status that is clear and understandable to the family. This information should be provided in a manner that is consistent with the family's ability to understand. This requires frequent and full updates and information exchanges between the staff members. Clear designation of topics and guidelines for family-staff discussions and staff training in communication with families in crisis, are strategies that could improve the current situation [11,17].

In the current study, satisfaction with the level of involvement in the decision-making process, amount of information, and appropriate time for decision making were found to be similar to Heyland et al.'s findings [4]. Most of the respondents felt supported during the decision-making process, were given an adequate amount of hope, and were satisfied with the level of healthcare the patients received. These findings correlate with the large study of the FS-ICU 34 questionnaire development [4]. This supports the validity of our version of the questionnaire and stresses the differences we found in our research.

One important difference in our study was that more than half of the respondents did not feel they had control over the care of their family member even though they were officially appointed to be the LG. Heyland et al. [8], conversely, found that most of the respondents felt they had good or some control. The apparent contradiction might be explained by the stress, anxiety, and even depression that families of ICU patients experience in this stressful situation, coupled with lack of knowledge and full understanding of the implications of their decisions for the patient, which make it difficult for family members to take over their relative's care [3,11].

Our study demonstrates that most LGs prefer that decisions be made by the medical staff after discussion with the family, a shared decision-making model. This is different from the autonomous model, where patients or LGs are expected to independently make decisions, or from the paternalistic model where decisions are made primarily by the medical staff. The respondents found it difficult to make decisions for their relatives, especially when the patient's values and wishes regarding invasive procedures are unclear or unknown. The majority of the respondents was not aware or was not sure of the patients’ preferences regarding invasive procedures, similar to the findings of Sviri et al. [9]. This makes the LGs' decision-making process even more complicated. It seems that most LGs prefer a shared decision-making approach as seen by high levels of agreement with items where there is a role for both family members and physicians, most probably because of the difficulties they face when required to make decisions for others. These difficulties include difficulty to process and understand the implications of acting or not acting, vague knowledge of the patients' actual wishes, unease regarding taking responsibility for another person's life, and the fear that their decisions will be a subject of family conflicts. Interestingly, in the LG situation here there is more than one type of shared decision making, within the family and between the LG and the medical staff. Our findings are consistent with previous reports showing that families of ICU patients preferred a shared decision-making approach [4,17,18].

Most of the respondents felt that the decision could be made without the need for an LG appointment. This preference is in contradiction to the current law in Israel. The present situation requires LGs, instead of their sick loved ones, to sign an agreement for performing or withholding procedures, a requirement that supports the autonomous approach and protects both patients and families from a paternalistic decision-making approach. The only option to overcome this obstacle without changing the law is to encourage the use of advanced medical directives or power or attorney. This will allow the medical staff to honor the patient’s preferences, without the need to appoint an LG (who may not know or feel comfortable complying with the patient's wishes). It is therefore recommended that the use of advanced directives and power of attorney be promoted in every possible encounter with patients in the primary health care settings, emergency departments, and upon hospital, nursing home, and rehabilitation department admissions. It should also be a part of a widely advertised campaign aimed at increasing awareness of the availability and importance of advanced directives in chronically ill patients. It is reasonable to presume that the same person that is appointed to be the LG could have been granted the power of attorney if the patient had been aware of that option at the appropriate time. However, as there was not a single case of advanced medical directives or power or attorney found in our study population, no comparisons could be made.

Our study is the first to report LGs' preferences. Our findings indicate that the LGs' unique situation includes many challenges especially related to staff–family communication and need for special support for family members, especially the LGs. Special care should be taken to provide the LGs and the patients’ families with updated information without contradictions. According to our findings it is important to perform regular evaluation of the current LG appointment procedure that has been practiced for more than 15 years. We suggest that the medical staff make an effort to explain, inform, and enable LGs to feel they are capable of making decisions on behalf of their loved ones, as part of the decision-making process. They should not on one hand feel they are forced to make a one-sided decision, while on the other hand not be subjected to paternalistic decision making by the medical team, all this without contradicting the requirements of current laws.

### Study limitations

This study is the first that focuses on LGs of ICU patients; therefore no direct comparison to other studies is available. The sample consisted of a limited number of participants, only Hebrew speakers at two institutions. The presence of anxiety or depression was not measured but may have affected the study results. Although no significant differences were found between the two study hospitals or across a variety of socio-demographic groups, caution should be taken when generalizing the study findings beyond the study setting. This need for caution emanates primarily from limited sample size and possible differences across hospitals and countries in routines and attitudes related to LGs. Moreover, the LG appointment is performed by different Courts of Law in different regions, and this could lead to variations in the LG appointment procedure. Since different countries have different legal approaches and legal systems, care should be taken when generalizing our findings with other healthcare systems. Therefore, there is a need for further assessment of current practices regarding LG appointment in both the healthcare and legal systems in Israel and abroad.

## Conclusions

The legal guardianship process includes difficulties, especially in the areas of communication with the medical staff and support mechanisms for family members. There is a need for improved communication between the medical staff and LGs, especially in providing more consistent and understandable information. Most LGs and families prefer a shared decision-making model rather than an autonomous or paternalistic model. These findings are not limited to a certain demographic group and can be considered universal. LGs’ shared decision-making preferences, unknown patient treatment preferences, and LGs’ sense of lack of control over treatment should encourage the evaluation of current practices. Care should be taken to provide for LGs and families the information required for decision making while complying with the limitations of current legislation. Promotion of the formulation of advanced directives and/or appointment of a power of attorney in accordance with the Law of the Dying Patient (2005) is advised. Further research is needed to find strategies for improving current practices.

## Competing interests

There are no financial or non-financial competing interests to declare.

## Authors’ contributions

MK, FDG, SS, and DMLinton planned the study and drafted the article. MK collected the data and wrote the manuscript. All authors reviewed the draft manuscript and read and approved the final manuscript.

## Authors’ information

Michael Kuniavsky is a Critical Care RN, Clinical Instructor, and Nursing Educator at Assaf HaRofeh Medical Center. At the time of the study he was also a master's degree student in Advanced Clinical Nursing in the Hebrew University and Hadassah Henrietta Szold School of Nursing.

Freda DeKeyser Ganz is the head of the master's degree program in nursing at the Hebrew University and Hadassah Henrietta Szold School of Nursing.

David M. Linton is professor of Medicine, head of the Medical ICU at the Hadassah-Hebrew University Medical Center.

Sigal Sviri is a senior physician in the Medical ICU Hadassah-Hebrew University Medical Center.

## Supplementary Material

Additional file 1 Attitudes of LGs regarding the decision making process (ADMAP) questionnaire and LGs’ responses. Click here for file
