# Supplementary material for: The legal guardians' dilemma: Decision making associated with invasive non-life-saving procedures
Source: Isr J Health Policy Res. 2012 Sep 24;1:36. doi: 10.1186/2045-4015-1-36 (PMC3467171; doi:10.1186/2045-4015-1-36)
Supplement: Additional file 1 — Attitudes of LGs regarding the decision making process (ADMAP) questionnaire and LGs’ responses. [file 2045-4015-1-36-S1.doc]

**Appendix A. Attitudes of LGs regarding the decision making process (ADMAP) questionnaire and LGs’ responses.**

| 5  Strong agreement | 4 | 3  Unsure | 2 | 1  Strong disagreement |  |  |
| --- | --- | --- | --- | --- | --- | --- |
| **50**  **78.5%** | 10  15.6% | 2  3.1% | 1  1.6% | 1  1.6% | N  % | 1. All family members agreed to perform invasive procedure |
| 1  1.6% | 4  6.2% | **17**  **26.6%** | **17**  **26.6%** | **25**  **39.1%** | N  % | 2. "I prefer to make the decision by myself" |
| 3  4.7% | 12  18.8% | **16**  **25%** | **14**  **21.9%** | **19**  **29.7%** | N  % | 3. "I prefer that the medical staff make the decision" |
| **42**  **65.6%** | **16**  **25%** | 3  4.7% | -  - | 3  4.7% | N  % | 4. "I prefer that the medical staff will make the decision after discussing with me" |
| **42**  **65.6%** | 13  20.3% | 4  6.2% | 3  4.7% | 2  3.1% | N  % | 5. My opinion regarding the invasive procedure would have remained the same if I were not appointed as LG |
| **20**  **31.2%** | **17**  **26.6%** | 10  15.6% | 7  10.9% | 10  15.6% | N  % | 6. We could reach the decision without LG appointment |
| 9  14.1% | -  - | **13**  **20.3%** | **15**  **23.4%** | **27**  **42.2%** | N  % | 7. If the invasive procedure was performed without acquiring my permission I would take a legal action against the hospital |
| **15**  **23.4%** | **15**  **23.4%** | **24**  **37.5%** | 8  12.5% | 2  3.1% | N  % | 8. I know what kind of treatments and procedures the patient would have agreed to undergo |
| **14**  **21.9%** | **20**  **31.2%** | **24**  **37.5%** | 4  6.2% | 2  3.1% | N  % | 9. When making the decision regarding the invasive procedure I took the patient's preferences into consideration |
